# Effectiveness of drug safety measures for reducing the incidence of adverse drug reactions: Post-hoc analysis of data from all-case surveillance of iguratimod using generalized estimating equations

**DOI:** 10.1371/journal.pone.0253513

**Published:** 2021-07-30

**Authors:** Kai Shibata, Akiko Yoshimura, Satoshi Ikeuchi, Mika Ishii

**Affiliations:** Clinical Planning and Development Department, Medical HQ, Eisai Co., Ltd., Tokyo, Japan; PLOS ONE, UNITED KINGDOM

## Abstract

A post-marketing study was performed on all patients who had started treatment with iguratimod, a conventional synthetic disease-modifying antirheumatic drug approved in Japan. During the study period, various safety measures were implemented to reduce risks. We investigated the frequency of adverse drug reactions before and after implementation of each safety measure to examine the preventive effect of these measures. Post-hoc analysis was performed using data from all-case surveillance of iguratimod. The subjects were all of the patients receiving iguratimod for whom safety information was obtained. To identify the time after starting administration when adverse drug reactions were most likely to occur, a generalized linear mixed-effect model was applied for the period from initiation of administration until occurrence of reactions in each patient. The mean incidence of adverse drug reactions per patient was compared before and after the implementation of safety measures by using generalized estimating equations based on a two-sided test, 95% confidence interval, and 5% significance level. The number of patients treated with iguratimod was not related to changes in the number of patients with adverse drug reactions. After implementing precautions regarding co-administration with warfarin and liver dysfunction, the estimated mean incidence rate of adverse drug reactions (95% confidence interval) decreased significantly to 0.73 (0.59–0.90) and 0.72 (0.55–0.94), respectively. Accordingly, some of the implementation of safety measures significantly reduced adverse drug reactions. The effectiveness of safety measures implemented during the all-case surveillance of iguratimod was evaluated, revealing that early implementation of safety measures decreased the incidence of adverse drug reactions.

## Introduction

In the pharmaceutical affairs of Japan, the re-evaluation period of a new drug ranges from 4 to 10 years, depending on its approval category [1]. During this period, the pharmaceutical company conducts a single cohort observational study and performs a comparative cohort observational study. In addition, using sources of secondary medical information such as insurance claims and the Diagnosis Procedure Combination database, a post-marketing database study and/or post-marketing clinical trial are conducted. Efficacy and safety data are collected, evaluated, and submitted to the regulatory agency [2–4]. Moreover, when a limited number of Japanese patients were enrolled in the clinical trials or when there was concern about serious adverse drug reactions (ADRs), implementing all-case surveillance from the time of product launch is often imposed as a condition of approval, and this is continued until the target number of patients is reached [5]. Since the year 2000, such all-case surveillances have been required for more products. During the period from 2006 through 2013, an all-case surveillance as approval condition was imposed for approximately 30% of all newly approved new chemical entities [6], and it was required for many anti-rheumatic drugs too.

Risk management guidelines were established in the US and EU in 2005, after which risk minimization plans were developed [7, 8]. The effectiveness of risk minimization measures based on such plans has been evaluated from the number of spontaneous reports and from studies of insurance claims databases [9]. In Japan, the preparation and submission of a risk management plan has been required for all products for which an NDA was submitted after April 2013 [10]. Accordingly, it is just over 5 years since the introduction of risk minimization measures in Japan and their effectiveness has not been investigated sufficiently.

Iguratimod is a conventional synthetic disease-modifying antirheumatic drug (csDMARD), which is approved in Japan. As phase 3 trials, one study was conducted to compare the efficacy and safety of iguratimod treatment in comparison salazosulfapyridine treatment [11, 12], and another study comparing the efficacy and safety of the combined use of iguratimod and MTX [13] was also conducted. The adverse events related to liver function and gastrointestinal disorders have been observed.

As part of the approval conditions, all patients with rheumatoid arthritis receiving iguratimod from September 2012 through April 2013 were investigated [14]. During this all-case surveillance study period, various safety measures were implemented to reduce the incidence of ADRs. Information on safety measures was disseminated via leaflets for healthcare professionals, posts on the company website, alerts from the relevant academic societies, and notifications released by the regulatory agency. ADRs occurring in all patients treated with iguratimod were collected during the all-case surveillance study period, and this complete patient database was used to compare the incidence of ADRs before versus after implementation of safety measures, in order to evaluate their effectiveness. The mean incidence of ADRs per patient was calculated before and after implementation of safety measures, and then was compared by using generalized estimating equations.

## Methods

### All-case surveillance study

This multicenter, prospective, observational, post-marketing surveillance study has been previously registered (NCT 01850966). Since the all-case surveillance study was conducted under unique conditions attached to the approval, its protocol was fixed immediately before the initiation of product marketing. Furthermore, since the all-case surveillance study was begun immediately after product marketing, the procedure within the founder needed for registration into the registry site was delayed, and the study was registered at the registry site after the start of patient enrollment into the study.

All patients were registered centrally, starting from the sale of iguratimod in September 2012 to April 2013. Each patient was followed-up for 52 weeks. If iguratimod treatment was ended or a subject who had begun taking iguratimod stopped visiting before 52 weeks, the observation ended. Data on adverse events (AEs) were collected throughout the 52-week study period, based on reports from each attending physician. All patients with rheumatoid arthritis who received treatment with iguratimod were registered in the study, totaling 2,747 patients. Excluding patients who did not return after the first visit, 2,666 patients were analyzed as the safety analysis set. The detailed design and overall result of all-case surveillance study have been reported previously [15].

### Safety assessment

The safety analysis set comprised all collected case report forms, except for those of patients who did not present after the first visit. AE data were collected based on the safety reports made by each attending physician and were coded using the Medical Dictionary for Regulatory Activities version 19.1. The definitions of AEs and ADRs were based on the International Conference on Harmonization Guideline E2A [16]and E2D [17]. AEs were categorized according to the preferred term (PT) and system organ class (SOC).

### Adverse reactions related to bleeding

Considering that bleeding-related adverse reactions can develop following combined warfarin and iguratimod administration, adverse reactions related to bleeding were defined. The definitions used in previously reported all-case study were applied [14]. Based on the stratified architecture of “haemorrhage (SMQ)” in Standardised MedDRA Query (SMQ), events whose sub-SMQs corresponded to Haemorrhage terms (excluding laboratory terms) or Haemorrhage laboratory terms (SMQ), and those observed when anemia was accompanied by elevation of the international normalized ratio, were defined as “bleeding or abnormal changes in clotting function parameters.”

### Classification of liver dysfunction

The definitions used in the previously reported all-case study results were used [14]. Among AEs collected in the all-case study, events related to liver function under MedDRA SOC and PT were defined as liver dysfunction. Alcohol-related, pregnancy-related and liver neoplasm-related liver disorders were excluded based on a Standardized MedDRA Query (SMQ) for liver disorder. Events related to interactions with warfarin were also excluded.

### Classification of gastrointestinal disorders

The definitions used in the previously reported all-case study results were used [14]. Among AEs collected in the all-case study, events related to the gastrointestinal tract (from the oral cavity to the rectum) under MedDRA SOC and PT were defined as gastrointestinal disorders.

### Safety measures

The following information on four important safety measures was issued during the study period. December 2012: “Caution regarding concomitant use of warfarin.” May 2013: “Contraindication to concomitant use of warfarin” / “Caution regarding liver dysfunction” (These were issued almost simultaneously, so analysis of both was done for the same time period.). August 2013: “Caution regarding gastrointestinal disorders” (especially peptic ulcer). February 2014: “Caution regarding interstitial pneumonia.”

In May 2013, the package insert of iguratimod was revised to include a description of the contraindications of its use with warfarin. This revision was performed because the Japanese authority, Ministry of Health, Labour and Welfare (MHLW), considered that restricting the use of iguratimod was not required, but precautions should be exercised during its use. A Blue Letter was distributed to notify healthcare professionals. In addition, information was uploaded to the websites of the regulatory agency and the marketing authorization holder as well as those of the relevant academic organizations. Moreover, it was checked whether the registered patients were using warfarin and if any hemorrhagic ADRs had occurred. To provide information about the other safety measures, leaflets for healthcare professionals were prepared and distributed.

### Statistical analysis

We applied a generalized linear mixed-effect model to the period from the start of iguratimod administration for each patient until the occurrence of ADRs to identify the time after starting administration when onset of ADRs was most likely. To investigate trends, estimated values were calculated before and after the peak incidence of ADRs, and the Wald test was performed. The estimated least squares mean and its difference were calculated for the mean incidence rate of ADRs before and after the peak incidence of ADRs, followed by the Wald test. The mean incidence of ADRs per patient before and after implementation of the safety measures was compared by using generalized estimating equations. The point estimate and confidence interval of the number of patients, number of ADRs, and mean incidence rate (number of ADRs/month) were calculated before and after implementation of each safety measure. The mean incidence rate (difference of the least squares mean) and confidence interval were also calculated, followed by the Wald test.

All analyses were performed with SAS system version 9.3 software (SAS Institute Inc., Cary, NC, USA). Except when otherwise indicated, all tests were performed with the level of significance set at 5% (two-sided). When interval estimation was performed, it was assumed to be two-sided, and the confidence coefficient was assumed to be 95%.

### Ethics statement

This all-case surveillance study was conducted in accordance with the Japanese Good Post-marketing Study Practice (GPSP) and the Ministry of Health, Labour and Welfare Guideline. The Japanese authority, Pharmaceuticals, and Medical Devices Agency was reviewed and approved the protocol. Under GPSP, data available at medical institutions are collected in a manner that ensures personal information cannot be identified. Accordingly, informed consent is not required, in principle. However, this surveillance study was reviewed if assessment by the ethics committee or Institutional Review Board of a clinical site was deemed to be necessary. This all-case surveillance study was registered with JAPIC CTI (JapicCTI-152782 and JapicCTI-132051) and ClinicalTrials.gov (NCT01850966).

## Results

### Incidence and tendency of adverse drug reactions onset timing

Performing an all-case surveillance study was a condition for approval of iguratimod. In order to ensure appropriate use of iguratimod, information about its product characteristics was provided and contracts for this study were executed with the participating doctors and medical institutions after review of eligibility.

**Fig 1:**
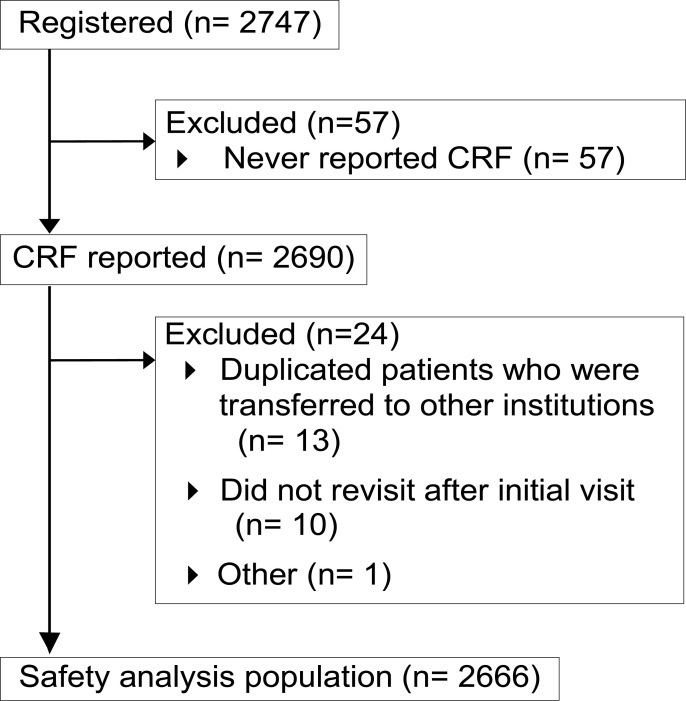
Study flowchart. The all-case surveillance study enrolled 2747 patients, and 2690 patients’ CRFs were collected. In this study, post-hoc analysis was conducted using the Safety analysis population (n = 2666) of the all-case surveillance study.

**Fig 2:**
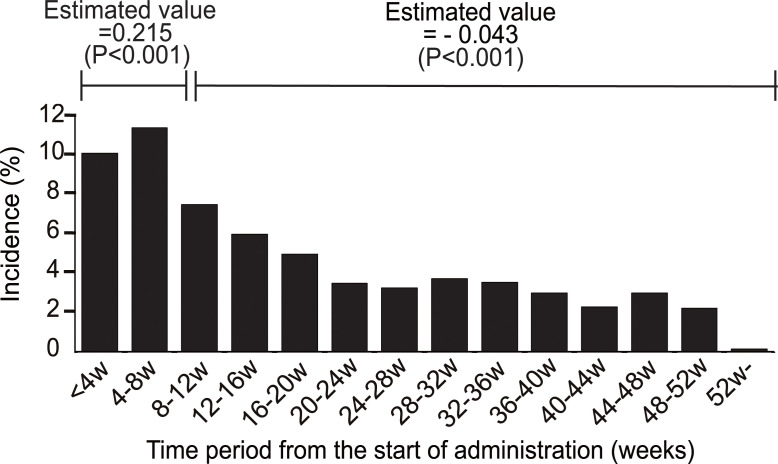
Incidence and tendency of ADRs onset timing. ADR trends for each patient The ADR incidence rate every 4 weeks from the start of administration is shown. The trends in the incidence rate were calculated from the start of administration up to 8 weeks and after 8 weeks with a generalized linear mixed-effect model, and estimated values are shown. The P value shown in the figure is from the Wald test. The incidence of ADRs by time period from the start of administration peaked after administration for 8 weeks and then showed a significantly decreasing trend.

This study used the all-case surveillance study database to compare the incidence of ADRs before versus after implementation of safety measures. The flowchart of this study is shown in Fig 1. The timing of the first onset of ADR in each registered patient has already been reported [15]. To confirm whether the trend was statistically different, a generalized linear mixed-effect model was tested. When the timing of onset for all ADRs was reviewed, a significant increase of reactions was found up to Week 8, followed by a subsequent significant decrease (Fig 2). The point estimate (95% confidence interval) of the difference in the mean incidence rate of ADRs (events/month) between before and after Week 8 was 0.52 (0.46–0.59).

### Change in the overall number of ADRs and implementation of safety measures

**Fig 3:**
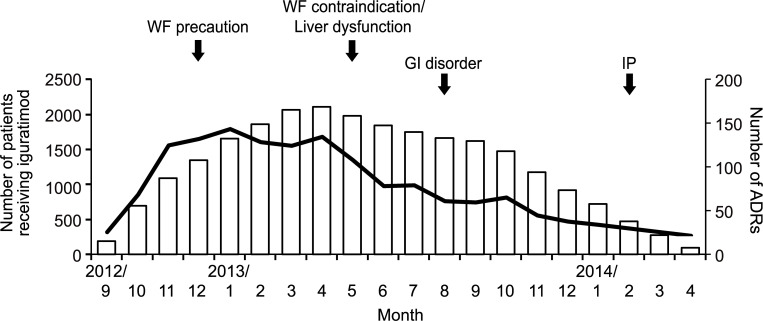
The overall number of ADRs and implementation of safety measures. The safety analysis was performed using the number of patients receiving iguratimod and the number of ADRs. The time when safety measures were implemented are shown by black arrows at the top. White bars show the number of patients receiving iguratimod each month, and the black line shows the number of ADRs per month during the study period are shown at the bottom. WF, warfarin; GI, gastrointestinal; IP, Interstitial pneumonia.

Since the rate of ADRs per patient was higher in the early phase of treatment, it was considered that the number of reactions would increase as the number of patients increased. The monthly number of patients on treatment with iguratimod showed a gradual increase during the study period until registration was terminated in April 2013 at the peak of enrollment, which was 2108 patients per month (Fig 3). Accordingly, the monthly number of ADRs was reviewed. The peak number was found to be 179 events/month in January 2013, which was earlier than the peak number of patients treated (Fig 3). The number of ADRs seemed to decrease around the timing of implementation of safety measures.

### Overall mean incidence rate of ADRs before and after implementation of safety measures

**Fig 4:**
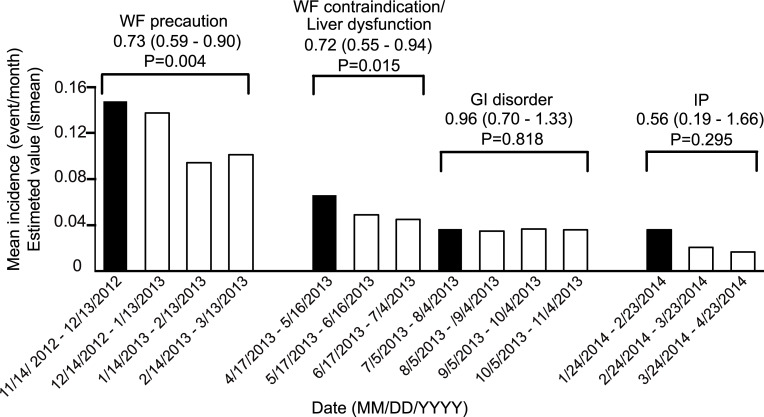
The incidence ratio of ADRs before and after safety measures. The estimated mean incidence rate (Lsmean) before and after safety measures was calculated, and the mean incidence ratio (difference in Lsmean) before and after the safety measures is shown in the upper graph. The black bars indicate before the safety measures, and the white bars indicate after the safety measures. The Wald test results are shown. WF, warfarin; GI, gastrointestinal; IP, Interstitial pneumonia.

To investigate whether the safety measures influenced the onset of ADRs, the incidence of ADRs per patient measures was compared before and after implementation of each safety measure. The point estimate (95% confidence interval) of the difference in the mean incidence rate of ADRs (events/month) before and after implementation was 0.73 (0.59–0.90) for the caution regarding co-administration of iguratimod with warfarin and 0.72 (0.55–0.94) for the cautions regarding warfarin and liver dysfunction. Thus, there was a significant decrease in ADRs after implementation of these safety measures (Fig 4 and Table 1). On the other hand, no significant decrease was observed after implementation of the other safety measures.

**Table 1:** The mean incidence ratio of overall ADRs.

			Mean incidence (event / month) (lsmean)					
		Date	Patient No.	Incidence No.	Estimated value	95% CI		
WF precaution	Before	2012/11/14–2012/12/13	1251	158	0.147	0.121	-	0.179
	After	2012/12/14–2013/01/13	1453	188	0.137	0.115	-	0.162
		2013/01/14–2013/02/13	1757	153	0.094	0.078	-	0.112
		2013/02/14–2013/03/13	1938	172	0.101	0.085	-	0.121
WF contraindication/ Liver dysfunction	Before	2013/04/17–2013/05/16	2026	142	0.066	0.054	-	0.081
	After	2013/05/17–2013/06/16	1914	99	0.049	0.039	-	0.061
		2013/06/17–2013/07/04	1795	50	0.045	0.033	-	0.061
GI disorder	Before	2013/07/05–2013/08/04	1733	71	0.037	0.028	-	0.049
	After	2013/08/05–2013/09/04	1662	64	0.035	0.027	-	0.047
		2013/09/05–2013/10/04	1609	60	0.037	0.028	-	0.048
		2013/10/05–2013/11/04	1429	51	0.036	0.026	-	0.052
IP	Before	2014/01/24–2014/02/23	525	17	0.036	0.021	-	0.064
	After	2014/02/24–2014/03/23	320	5	0.021	0.008	-	0.059
		2014/03/24–2014/04/23	152	1	0.017	0.002	-	0.123
		2014/04/24–2014/05/23	0	0	-	-	-	-

WF, Warfarin; GI, Gastrointestinal; IP, Interstitial pneumonia; CI, confidential interval.

The following is a detailed description of the safety measures for concomitant use with warfarin. The caution regarding concomitant use with warfarin was issued in December 2012 because of reports of serious bleeding or abnormal changes in blood coagulation test (increased PT-INR) that were suspected to be due to an interaction between iguratimod and warfarin. Then, due to a case of death caused by concomitant use with warfarin, an safety measure about contraindication to concomitant use of warfarin was issued in May 2013.Forty patients received iguratimod in combination with warfarin. Of 40 patients, 65.00% (26/40 patients) developed adverse reactions, the incidence of bleeding or abnormal changes in clotting function parameters was 47.50% (19/40 cases). The adverse reactions expressed using PTs were international normalized ratio increased, prothrombin time increased, subcutaneous haemorrhage, anaemia, conjunctival haemorrhage, hematoma, epistaxis, alveolar haemorrhage, gingival haemorrhage, melena and puncture site harmorrhage. Although in the interim analysis, comprehensive data on the 18 patients with hemorrhage-related ADRs have been reported [14].

### Mean monthly incidence rate of ADRs (Liver dysfunctions and gastrointestinal disorders)

**Fig 5:**
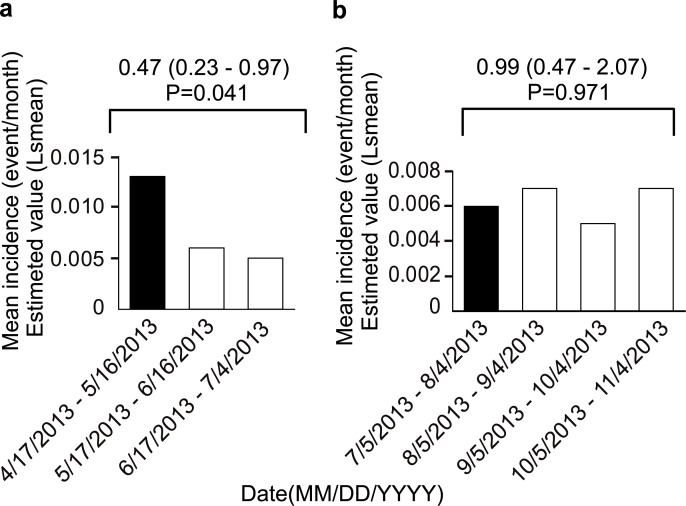
The mean incidence ratio of specific ADRs. a) Liver dysfunction, as an ADR, showed a significant decrease in the incidence rate after the precaution regarding liver dysfunction was issued. b) GI dysfunction, as an ADR, showed no change in the incidence rate before and after the precaution regarding GI dysfunction was issued. The black bars indicate before the safety measures and the white bars indicate after the safety measures. The Wald test results are shown.

During the all-case surveillance study period, safety measures were issued for specific ADRs that were identified as risks, so whether the incidence of specific ADRs decreased after implementation of safety measures was investigated. The most common ADRs during the all-case surveillance study period were gastrointestinal disorders (10.43%) and liver dysfunctions (9.71%) [15], so these two ADRs were investigated in detail. According to the safety measure for gastrointestinal disorders, although it was focused on pepticulcer, this analysis was performed using overall gastrointestinal disorders. Because endoscopic examination is not performed much in routine practice for rheumatoid arthritis or several physicians seem to have recognized the safety measure for overall gastrointestinal disorders. The point estimate (95% CI) of the difference in the mean incidence of liver dysfunction before and after implementation of safety measures for liver dysfunction was 0.47 (0.23–0.97). Similarly, the point estimate (95% CI) of the difference in the mean incidence of gastrointestinal disorders before and after implementation of safety measures for these disorders was 0.99 (0.47–2.07). A significant decrease in ADRs was noted after implementation of the caution for liver dysfunction (Fig 5 and Table 2).

**Table 2:** The mean incidence ratio of Liver dysfunction or GI disorder.

Date			Mean Incidence (event / month) (lsmean)					
			Patient No.	Incidence No.	Estimated value	95% CI		
WF contraindication/Liver dysfunction	Before	2013/04/17–2013/05/16	2026	27	0.013	0.009	-	0.019
	After	2013/05/17–2013/06/16	1914	13	0.006	0.003	-	0.014
		2013/06/17–2013/07/04	1795	6	0.005	0.002	-	0.013
GI disorder	Before	2013/07/05–2013/08/04	1733	12	0.006	0.003	-	0.012
	After	2013/08/05–2013/09/04	1662	12	0.007	0.004	-	0.012
		2013/09/05–2013/10/04	1609	9	0.005	0.003	-	0.01
		2013/10/05–2013/11/04	1429	10	0.007	0.004	-	0.013

WF; Warfarin, GI; Gastrointestinal, CI; confidential interval.

## Discussion

This study showed that the change in the number of ADRs was not related to the number of patients on treatment with iguratimod. Instead, implementation of safety measures decreased the incidence of ADRs, and our review of clinical data revealed that pharmaceutical company activities promoting appropriate use of iguratimod were effective in reducing ADRs. This is the first report on the effectiveness of new safety measures based on data obtained by all-case surveillance. The present study also indicated that implementation of precautions by a pharmaceutical company worked to decrease the incidence of ADRs related to a specific risk.

The incidence of ADRs to iguratimod was highest immediately after product launch, followed by a gradual decline. A high incidence of ADRs immediately after release of a new product followed by a subsequent decline is known as the Weber effect, and it is considered to occur because energetic detection of ADRs to new products results in an increase in reports [18]. This phenomenon was also identified by a study of spontaneous reports in the US, but other events might have influenced the outcomes in that study since the data came from spontaneous reports [19]. In the present study, although it was found that the incidence of events was higher immediately after the launch of iguratimod and then gradually decreased, the number of ADRs decreased significantly after the implementation of safety measures. The Dear Healthcare Professional Letters of Rapid Safety Communications (Blue Letter) was issued by the regulatory agency concerning the contraindication to use of iguratimod with warfarin, and this might have had a greater impact on healthcare professionals. The incidence of liver dysfunction also decreased after implementation of safety measures, possibly because of appropriate treatment adjustment in patients susceptible to the ADR and earlier initiation of preventive measures. Based on these findings, the changes in iguratimod-related ADRs during the all-case surveillance study period reflected the effectiveness of the safety measures implemented.

In contrast, implementation of safety measures for gastrointestinal disorders and interstitial pneumonia did not lead to a significant change in the mean incidence rate of ADRs. The safety measures for gastrointestinal disorders were mainly precautions related to gastrointestinal ulcers. During the all-case surveillance study, the incidence of gastrointestinal ulcers was 1.61% (29/1797) in patients co-administered NSAIDs, and they accounted for the majority of patients who developed such ADRs [14, 15]. Gastrointestinal disorders linked to NSAIDs have already been studied in patients with rheumatoid arthritis, and risk factors for Japanese patients have been identified [20]. According to the Japan College of Rheumatology Guideline, COX2-selective NSAIDs are associated with lower incidence of gastrointestinal ulcers than non-selective NSAIDs [21]. Thus, measures have already been implemented in Japanese clinical setting to prevent gastrointestinal disorders in patients co-administered NSAIDs, which may explain why a significant decrease of ADRs was not noted in this study. Also, the safety measures for gastrointestinal disorders and interstitial pneumonia were implemented in the late phase of all-case surveillance study. Since these precautions were not implemented during the early phase when the incidence was high, their impact might have been reduced. However, leaflets were also distributed to patients to communicate precautions about gastrointestinal disorders linked to NSAIDs at that time, physicians potentially interviewed patients receiving iguratimoid treatment about signs of gastrointestinal bleeding in the course of routine clinical care. Physicians could have also taken other measures during routine clinical care, such as the periodic monitoring of the levels of KL-6 (an indicator of interstitial pneumonia).

This study evaluated the effectiveness of risk minimization measures by implementing precautions based on Case Report Forms collected from all patients, so the evaluation was based on clinical primary data. The effectiveness of risk minimization is often investigated based on insurance claims databases, although a study has been performed that utilized data collection by questionnaire [22]. Contrary to the data from spontaneous reports of ADRs, all-case surveillance study provides information on the total number of patients exposed at a given time point. Using this feature, the incidence per patient was calculated on the basis of the number of patients who continued to receive the medication in this study. The number of adverse reactions was counted on the basis of the onset day. Therefore, whether or not iguratimod treatment was continued after the onset of adverse reactions did not affect the incidence of adverse reactions, thus enabling a valid comparison of the number of new adverse reactions. Furthermore, when using secondary sources such as insurance claims databases, there are problems with definitions of ADRs, and some reactions need validation. On the other hand, data obtained from all-case surveillance study are based on medical records and all ADRs could be collected. However, if a risk minimization measure is implemented during the late phase of all-case surveillance, its effectiveness cannot be investigated thoroughly. After the completion of the all-case surveillance study, the two precautions were issued, that is, in February 2014 (Caution regarding interstitial pneumonia) and January 2017 (addition of agranulocytosis in the package insert). The effects of adding the safety measures cannot be evaluated from the data of the all-case surveillance study. Longer-term observational data may be necessary. Moreover, we could not evaluate clinical laboratory abnormalities since an observational study cannot intervene to do clinical laboratory test. It is necessary to use other data sources to investigate these aspects.

In conclusion, we were able to use data obtained from all-case surveillance study of iguratimod in Japan to assess the effectiveness of safety measures implemented to decrease the incidence of ADRs during the all-case surveillance period. We found that safety measures implemented during the early phase of surveillance were effective for decreasing the incidence of ADRs.

## Supporting information

S1 Checklist Trend statement checklist. (PDF)

S1 File (PDF)

S2 File (PDF)
