# Supplementary material for: Effectiveness of drug safety measures for reducing the incidence of adverse drug reactions: Post-hoc analysis of data from all-case surveillance of iguratimod using generalized estimating equations
Source: PLoS One. 2021 Jul 30;16(7):e0253513. doi: 10.1371/journal.pone.0253513 (PMC8323896; doi:10.1371/journal.pone.0253513)
Supplement: S1 File — (PDF) [file pone.0253513.s002.pdf]

第 7.0 版：平成 27 年 6 月 5 日作成

ケアラム®錠 25mg 特定使用成績調査（長期）実施計画書
－　関節リウマチに対する安全性・有効性の検討（全例調査）－

1. 調査の目的

本剤の投与症例全例を対象として，使用実態下における本剤投与開始後 52 週までの安全性及び有効性を検討し，以下の事項を把握する。特に，高齢者又は低体重者での安全性，肝機能障害を発現した症例の予後，NSAID との併用による安全性（特に消化性潰瘍の発現状況）についても把握する。

1. 未知の副作用
2. 使用実態下における副作用の発現状況
3. 安全性又は有効性に影響を与えると考えられる要因

【重点調査項目】
- 血液障害の発現状況
- 肝機能障害の発現状況
- 腎機能障害の発現状況
- 胃腸障害（消化性潰瘍等）の発現状況
- 間質性肺炎の発現状況
- 感染症の発現状況

2. 調査を予定する症例数及び設定根拠

2.1 調査予定症例数

ケアラム®錠，コルベット®錠を合算して 2,000 例

発売開始から本剤が使用された全例の登録を行い，調査予定症例数である 2,000 例の調査票 1(投与開始から 24 週まで) について集計解析を実施して，厚生労働省に承認条件解除に関する要望書を提出する。なお，承認条件の解除までの間は，患者の登録は継続し，必要に応じ調査票を回収する。また，24 週を超えて投与継続された症例については，調査票 2（25 週から 52 週後まで）の収集も行い，集計解析を実施する。

2.2 設定根拠

開発時に 0.5%以上で発現した副作用が，使用実態下でも同様の発現頻度であることを確認するために，95%の確率で少なくとも 5 例検出するには 1,831 例必要である。そのため，近似値としてケアラム®錠，コルベット®錠を合算して 2,000 例と設定した。

3. 調査の対象となる患者

3.1 対象患者

関節リウマチを有する患者

＜警告＞
海外の臨床試験において，1 日 125 mg を投与した症例で致命的な転帰に至った汎血球減少症が認められている。本剤は緊急時に十分な措置が可能な医療施設において，本剤についての十分な知識とリウマチ治療の経験をもつ医師が使用すること。

＜禁忌（次の患者には投与しないこと）＞
1. 妊婦又は妊娠している可能性のある婦人
〔動物実験 (ラット) で，催奇形性，早期胎児死亡率の増加及び胎児の動脈管収縮が認められている〕
2. 重篤な肝障害のある患者
〔副作用として肝機能障害があらわれることがあるので，肝障害を更に悪化させるおそれがある〕
3. 消化性潰瘍のある患者
〔副作用として消化性潰瘍があらわれることがあるので，消化性潰瘍を更に悪化させるおそれがある〕
4. 本剤の成分に対し過敏症の既往歴のある患者
5. ワルファリンを投与中の患者

＜慎重投与（次の患者には慎重に投与すること）＞
(1) 授乳婦
(2) 肝障害又はその既往歴のある患者
［肝障害を更に悪化又は再発させるおそれがある］
(3) 消化性潰瘍の既往歴のある患者
［消化性潰瘍を再発させるおそれがある］
(4) 低体重の患者
(5) 貧血，白血球減少症，血小板減少症を伴う患者，骨髄機能低下患者
［血液障害を更に悪化させるおそれがある］
(6) 腎障害のある患者
［副作用の発現が増加するおそれがある］

＜ケアラム®錠 25 mg 添付文書（第 4 版）＞

3.2 用法・用量

通常，成人にはイグラチモドとして 1 回 25 mg を 1 日 1 回朝食後に 4 週間以上経口投与し，それ以降，1 回 25 mg を 1 日 2 回 (朝食後，夕食後) に増量する。

＜用法及び用量に関連する使用上の注意＞
1. 1 日 50 mg から開始した場合，1 日 25 mg の場合と比較して，AST (GOT)，ALT (GPT) 増加の発現率が高かったため，投与開始から 4 週間は 1 日 25㎎ を投与すること。
2. 1 日 50 mg を超えて投与しないこと。
3. 本剤の効果は，通常，投与開始後 16 週までに発現するので，16 週までは継続投与し，効果を確認することが望ましい。
4. 本剤並びに疾患の特性を考慮して，治療にあたっては経過を十分に観察し，漫然と投与を継続しないこと。

＜ケアラム®錠 25 mg 添付文書（第 4 版）＞

4. 調査を予定する診療科別の施設数

本剤を使用する全ての施設・診療科において調査を実施する。なお，本剤の使用は，以下の施設要件，医師要件を満たし，本調査の契約を締結した施設及び医師に限定する。

(1)施設要件

以下の全ての要件を満たす施設を対象とする。

1)全例調査への協力ならびに契約が可能な施設

2)関節リウマチの専門治療が可能な施設 (T-614 の治験参加施設，日本リウマチ学会認定教育施設，日本整形外科学会専門医研修施設　等)

3)緊急時に十分な措置が可能な施設

(2)医師要件

以下のいずれかの要件を満たす医師で，本剤の適正使用情報の説明を受け，本剤の関節リウマチへの治療について十分理解された医師を対象とする。

1)T-614 の治験参加医師

2)日本リウマチ学会専門医

3)日本整形外科学会認定リウマチ医

4)日本リウマチ財団登録医

5. 調査の方法

本調査は中央登録方式の全例調査とする。

5.1 調査の依頼・契約

1)医薬情報担当者が本剤の適正使用情報及び特徴，本調査の目的，調査対象，調査方法等を調査担当医師等に説明する。

2)調査担当医師等は，適正使用情報の説明を受けたこと，製造販売後調査へ協力すること，施設要件及び医師要件を満たしていることを確認し，「適正使用情報確認書」に署名する。医薬情報担当者は，調査担当医師等により署名された「適正使用情報確認書」を入手，調査実施施設及び調査担当医師の要件を満たしていることを確認後，本調査を施設に依頼し，医療機関の長と文書による契約を締結する。

3)施設との本調査の契約締結後に，本剤を納入する。

5.2 調査対象患者の選定方法及び症例登録

1)本剤の発売開始日から承認条件が解除されるまで本剤を使用する全例を登録する。

2)調査担当医師は，本剤投与開始後，14 日以内を目処に登録票に必要事項を記入し，登録センターに FAX 送信することによって症例登録を行う。

5.3 観察期間

1 例あたりの観察期間は，本剤投与開始から 52 週間とする。

5.4 調査票の記載及び提出

1)本調査の調査内容の収集手段として，紙面による調査票を用いる。調査票は 2 分冊とする。投与開始から 24 週以内に本剤を中止された症例は，調査票 1 で終了とする。ただし，投与開始から 52 週後までに投与を再開する場合は，継続して調査票にて情報を収集する。

また，有害事象が発現した場合は，観察期間終了後も当該事象が回復するまで検査/観察を継続する。

【調査票】　調査票 1：投与開始時～24 週後
調査票 2：25 週～52 週後

2)調査担当医師は，分冊毎の観察期間終了時点で，観察結果に基づき，速やかに調査票に必要事項を記入し，医薬情報担当者へ提出する。

3)調査依頼者は，登録票及び調査票の記載内容を確認し，必要に応じて再調査を実施する。

5.5 全例登録の確認

登録例数が調査予定症例数に到達した時及び調査終了時に，調査依頼者は調査担当医師等から「全例登録確認書」を入手することにより，本剤を投与された患者全例が登録されていることを確認する。

5.6 中止症例について

調査担当医師は本剤の投与を中止した症例について，調査票に中止日，中止理由を記載し，中止時点までの評価を記載する。ただし，投与開始から 52 週後までに投与を再開する場合は，継続して調査票にて情報を収集する。

6. 調査の実施予定期間

調査実施予定期間は，以下のとおりとする。

調査期間　発売開始～承認条件解除，又は，観察期間（52 週）終了のいずれか遅い方
登録は厚生労働省により承認条件が解除されるまで継続する。

7. 調査を行う事項等

7.1 調査を行う事項

(1)登録票

登録票には下記の項目を記入する。

- 禁忌症例に該当しないことの確認
- 慎重投与項目の確認
- 投与前臨床検査実施の確認
- 患者イニシャル
- 患者識別コード
- 性別（妊娠・授乳の有無）
- 生年月日又は年齢
- 使用理由
- 関節リウマチの罹病期間
- 関節リウマチの病期・進行度(Steinbrocker の病期分類)[1)](**エラー! 参照元が見つかりません。**)
- 関節リウマチの機能障害度(Steinbrocker の機能分類)[1)](**エラー! 参照元が見つかりません。**)
- 投与開始時の肝機能障害
- 投与開始時の腎機能障害
- 直近の胸部画像検査
- 直近の B 型肝炎ウイルス検査
- 投与開始日

なお，妊娠・授乳ありの場合は，調査票入手後に追跡調査を行う。

(2)調査票

調査票には下記の項目を記入する。

1)患者背景

・体重
・関節リウマチに対する手術歴
・抗 CCP 抗体
・既往歴
・投与開始時の受診区分
・リウマトイド因子
・合併症
・アレルギー歴

2)本剤の投与経過

①本剤の選択理由 [調査票 1]

②本剤の投与状況 [調査票 1，調査票 2]
1 日量，投与期間，用法・用量外の使用理由

③24 週後/52 週後の本剤の投与状況 [調査票 1，調査票 2]
継続，中止
中止した場合は，その理由

3)本剤以外のリウマチ治療薬 (3 ヵ月以内の前治療薬及び併用薬：生物学的製剤・DMARD・ステロイド) [調査票 1，調査票 2]
本剤以外のリウマチ治療薬 (3 ヵ月以内の前治療薬及び併用薬：生物学的製剤・DMARD・ステロイド) の有無，薬剤名，投与経路（ステロイドのみ），投与量，投与期間

4)その他の併用薬剤 (NSAID を含む) [調査票 1，調査票 2]
その他の併用薬剤 (NSAID を含む) の有無，薬剤名，投与経路，使用理由
なお，有害事象が発現した場合のみ，投与量及び投与期間を記入する。

5)関節リウマチに対する併用療法 [調査票 1，調査票 2]
関節リウマチに対する併用療法の有無，種類

6)臨床経過 (関節リウマチの症状・所見) [調査票 1，調査票 2]

・圧痛関節数（28 関節）
・ESR (1 時間値)
・患者による全般評価 (VAS)
・腫脹関節数（28 関節）
・CRP
・担当医師による全般評価(VAS)

7)重点調査項目に関する検査値及び所見 [調査票 1，調査票 2]
以下の重点調査項目について確認する。本剤投与中に血液学的検査値，肝機能検査値，腎機能検査値の異常変動，胃腸障害（消化性潰瘍等），間質性肺炎，感染症を疑う症状/検査異常が認められた場合は，その所見を有害事象欄に記入する。臨床検査値の異常変動の有無は，**エラー! 参照元が見つかりません。**の基準を参考に判定する。ただし，患者の生理変動内の変化，本剤投与前から持続し悪化を伴わない異常値は異常変動としない。

<table>
<tr><th rowspan="2">重点調査項目</th><th rowspan="2">検査/観察の時期</th><th colspan="2">検査/観察項目</th></tr>
<tr><th>全症例</th><th>有害事象発現時</th></tr>
<tr><td>血液障害</td><td rowspan="3">投与開始時,
2 週後, 4 週後,
6 週後, 8 週後,
12 週後, 16 週後,
20 週後, 24 週後,
28 週後, 32 週後,
36 週後, 40 週後,
44 週後, 48 週後,
52 週後
又は投与中止時</td><td>白血球数
白血球分画
赤血球数
ヘモグロビン量
ヘマトクリット値
血小板数</td><td>骨髄検査
白血球数
白血球分画
赤血球数
ヘモグロビン
ヘマトクリット
血小板数 等</td></tr>
<tr><td>肝機能障害</td><td>AST（GOT）
ALT（GPT）
ALP
LDH
γ-GTP
総ビリルビン
アルブミン</td><td>AST（GOT）
ALT（GPT）
ALP
LDH
γ-GTP
総ビリルビン
アルブミン 等</td></tr>
<tr><td>腎機能障害</td><td>BUN
血清クレアチニン
尿中 NAG
尿中 β_2-ミクログロブリン
尿検査（尿蛋白，尿糖，尿潜血，尿沈渣）</td><td>BUN
血清クレアチニン
尿中 NAG
尿中 β_2-ミクログロブリン
尿検査（尿蛋白，尿糖，尿潜血，尿沈渣）等</td></tr>
<tr><td>胃腸障害（消化性潰瘍等）</td><td rowspan="3">投与開始から
投与中止まで</td><td>発症もしくは増悪を疑う症状／検査異常</td><td>内視鏡検査
出血</td></tr>
<tr><td>間質性肺炎</td><td rowspan="2">KL-6（投与開始時）
発症もしくは増悪を疑う症状／検査異常</td><td rowspan="2">胸部画像所見
感染症の原因に関する検査
白血球数
白血球分画
CRP
KL-6
β-D グルカン 等</td></tr>
<tr><td>感染症</td></tr>
</table>

8)有効性 [EULAR DAS28 による臨床効果判定[2)] (エラー! 参照元が見つかりません。)]

各判定時に，以下の手順で臨床効果を判定する。なお，判定は調査依頼者が調査票回収後に一括して行う。

ｉ）DAS28 の算出評価

a) 関節の評価 (圧痛関節数，腫脹関節数)，b) ESR (1 時間値) 又は CRP，c) 患者による全般評価 (VAS) から，計算式により算出する。測定時点の DAS28 は下記のように分類する。

1．DAS ≤3.2　　2．3.2< DAS ≤ 5.1　　3．DAS > 5.1

ⅱ）DAS28 改善度

投与開始時の DAS28 から各判定時の DAS28 の差を算出し，DAS28 改善度を下記のように分類する。

1．差 > 1.2　　2．0.6 < 差 ≤ 1.2　　3．差 ≤ 0.6

ⅲ）DAS28 による臨床効果判定

各判定時のⅰ) DAS28 算出評価とⅱ) DAS28 改善度から EULAR DAS28 改善度による効果判定に基づいて臨床効果を下記の 3 段階で判定する。

1．有効　　2．やや有効　　3．無効

9)安全性

本剤投与中に発現した全ての好ましくない又は意図しない疾病又はその症状，徴候，あるいは臨床検査値等の異常変動を有害事象とし，本剤との因果関係の有無は問わない。ただし，自然経過による関節リウマチの悪化は有害事象として取り扱わない。なお，有害事象のうち，本剤との因果関係が否定できないものを副作用とする。

観察期間終了時までに発現した有害事象について，以下の項目を調査する。なお，有害事象は原則として，調査担当医師が臨床的に問題ないと判断するまで経過観察を行う。また，重篤な有害事象や未知の事象が発現した場合は，詳細な調査を行う。

有害事象の有無，有害事象名，発現日，重症度，重篤性，重篤理由，有害事象発現に対する本剤の投与状況，有害事象に対する治療，転帰日（又は転帰確認日），転帰，本剤との因果関係，本剤以外の要因，重点調査項目に関連する検査（有害事象発現時），重点調査項目以外の臨床検査値，有害事象に関するコメント

10)ワルファリンを含む抗凝固薬との相互作用に関する検討

本剤とワルファリンとの相互作用が疑われる出血又は血液凝固能検査値の異常変動 (PT-INR 増加) が報告されたことから，2013 年 5 月にワルファリンとの併用を禁忌とした。本措置に伴い，調査票において，ワルファリン併用症例及びワルファリン以外の抗凝固薬併用症例を把握した場合は，それぞれ以下の情報を収集する。

1)　ワルファリン併用症例

出血事象発現の有無，出血事象が発現した場合はその詳細情報，ワルファリンの投与量，本剤との併用前後の血液凝固系臨床検査値 (PT-INR)

2)　ワルファリン以外の抗凝固薬併用症例

出血事象発現の有無，出血事象が発現した場合はその詳細情報，抗凝固薬の投与量，本剤との併用前後の血液凝固系臨床検査値 (PT-INR, APTT 等)

7.2 調査スケジュール

<table>
<tr><td colspan="2">調査票　分冊番号</td><td colspan="4">調査票 1</td><td colspan="2">調査票 2</td></tr>
<tr><td colspan="2">時期
項目</td><td>投与開始時</td><td>2　8
週〜週
後　後</td><td>12　20
週〜週
後　後</td><td>24投
週与
後中
又止
は時</td><td>48
〜週
後</td><td>52投
週与
後中
又止
は時</td></tr>
<tr><td colspan="2">患者背景</td><td>●</td><td></td><td></td><td></td><td></td><td></td></tr>
<tr><td colspan="2">本剤の投与経過</td><td>←</td><td>―</td><td>―</td><td>―</td><td>―</td><td>→</td></tr>
<tr><td colspan="2">本剤以外のリウマチ治療薬</td><td>←</td><td>―</td><td>―</td><td>―</td><td>―</td><td>→</td></tr>
<tr><td colspan="2">その他の併用薬剤</td><td></td><td>←</td><td>―</td><td>―</td><td>―</td><td>→</td></tr>
<tr><td colspan="2">関節リウマチに対する併用療法</td><td></td><td>←</td><td>―</td><td>―</td><td>―</td><td>→</td></tr>
<tr><td colspan="2">臨床経過
28 関節評価（圧痛・腫脹）
ESR，CRP，
患者による全般評価（VAS）
担当医師による全般評価（VAS）</td><td>●</td><td>●
（4 週毎）</td><td>●
（4 週毎）</td><td>●</td><td>●
（4 週毎）</td><td>●</td></tr>
<tr><td rowspan="2">重点調査項目</td><td>血液障害
肝機能障害
腎機能障害</td><td>●</td><td>●
（2 週毎）</td><td>●
（4 週毎）</td><td>●</td><td>●
（4 週毎）</td><td>●</td></tr>
<tr><td>胃腸障害（消化性潰瘍等）
間質性肺炎
感染症</td><td></td><td>←</td><td>―</td><td>―</td><td>―</td><td>→</td></tr>
<tr><td colspan="2">有害事象の確認</td><td></td><td>←</td><td>―</td><td>―</td><td>―</td><td>→</td></tr>
</table>

投与中止時は，その時点での必要な観察・検査及び評価を実施する。ただし，投与開始から 52 週後までに投与を再開する場合は，観察・検査及び評価を継続する。

8. 解析を行う項目及び方法

8.1 症例の取り扱い

症例の採否，判定及び有害事象については，以下の基準に従う。取り決めがない事項が発生した場合は，症例検討会にてその取り扱いを決定する。

8.1.1 安全性判定

以下の症例は安全性解析対象から除外する。

(1)本剤が投与されていない症例

(2)初回処方日以降に来院しなかった症例
(3)過去に本調査に登録されたことがある症例

8.1.2 有効性判定

安全性解析対象のうち以下の症例は有効性解析対象から除外する。
(1)対象外疾患の症例
(2)投与開始時に DAS28 算出に必要な項目が欠測している症例
(3)調査開始前より本剤を投与されていた症例 (製造販売後臨床試験からの継続症例)

8.1.3 その他の取り扱い基準

データ範囲の詳細は解析計画書で規定する。

8.2 解析項目

解析は以下に示す項目を実施する。なお，その他の解析が必要な場合は，その必要性，科学性を検討した上で，追加解析を行う。

8.2.1 症例構成に関する事項

調査施設数，登録症例数，調査票回収症例数，安全性解析対象症例数，有効性解析対象症例数，解析除外例数及び除外理由　等

8.2.2 安全性に関する事項

副作用発現率，背景因子別副作用発現率，重篤な副作用発現率，有害事象発現率，重篤な有害事象発現率，有害事象発現症例一覧表 等

8.2.3 有効性に関する事項

DAS28 による臨床効果，背景因子別臨床効果 等

8.2.4 重点調査項目

血液障害，肝機能障害，腎機能障害，胃腸障害（消化性潰瘍等），間質性肺炎，感染症の副作用発現率　等

8.2.5 特別な背景を有する患者に関する事項

肝機能障害を有する患者，腎機能障害を有する患者，小児，高齢者，低体重 (40 kg 未満)の患者

8.3 解析方法

解析方法は，比較をする場合は χ^2 検定，Fisher 直接確率法，t 検定等の手法から項目に応じて適切な解析を実施する。

一方，本調査の安全性及び有効性に影響を及ぼす背景要因を検討する場合はロジスティック回帰分析等を実施する。

8.3.1 安全性に関する事項

(1)副作用について，発現症例数及び発現症例率，種類別の発現件数及び発現率を算出する。
また，重篤な副作用について，発現症例数及び発現症例率，種類別の発現件数及び発現率を算出する。
(2)安全性に影響を与えると考えられる要因を検討するために，患者背景別の副作用発現率を算出するとともに，$\chi 2$ 検定あるいは Fisher 直接確率法を行う。有意水準は両側 5%とする。

(3)有害事象について，発現症例数及び発現症例率，種類別の発現件数及び発現率を算出する。また，重篤な有害事象について，発現症例数及び発現症例率，種類別の発現件数及び発現率を算出する。

8.3.2 有効性に関する事項

(1)臨床効果判定は，EULAR の DAS28 改善度で「有効」又は「やや有効」と判定された症例を有効として集計する。

(2)有効性に影響を与えると考えられる要因を検討するために，患者背景別の有効率を算出するとともに，χ^2 検定あるいは Fisher 直接確率法を行う。有意水準は両側 5%とする。

8.3.3 その他の事項

腎機能障害の分類は，投与開始時の血清クレアチニン及び体重より Cockcroft-Gault 式を用いクレアチニンクリアランス CLcr (mL/min) を算出し，FDA ガイダンス (Guidance for Industry : Pharmacokinetics in Patients with Impaired Renal Function –Study Design, Data Analysis, and Impact on Dosing and Labeling) に基づいて，正常腎機能 ($CLcr \geq 80$)，軽度腎機能障害者 ($50 \leq CLcr < 80$)，中等度腎機能障害者 ($30 \leq CLcr < 50$)，重度腎機能障害者 ($CLcr < 30$) に分類する。

9. 調査実施のための組織体制

「医薬品リスク管理計画書」に記載した。

10. その他必要な事項

(1)実施計画書の改訂

本調査の実施期間中に，新たな知見が得られた場合，実施計画書の改訂の要否を検討し，必要に応じて改訂を行うこととする。また，本調査実施中に用法・用量あるいは効能・効果の一部変更承認（新規の再審査期間が指定された場合を除く）等を受けた場合にも，実施計画書の改訂の要否を検討し，必要に応じて改訂を行うこととする。

(2)問題点，疑問点が認められた場合の処置

以下の場合には，それら要因を検出又は確認するため，また検討を行った結果得られた推定等を検証するために，新たな特定使用成績調査，製造販売後臨床試験の実施を検討する。

1)未知・重篤の副作用の発現等の重大な安全性の懸念が示唆された場合
2)副作用発現頻度の明らかな上昇がみられた場合
3)承認時までの試験結果と比較し，安全性及び有効性に何らかの問題点が見出された場合

引用文献

1 Steinbrocker O.et al.Therapeutic criteria in rheumatoid arthritis.JAMA140:659, 1949

2 Department of rheumatology, University Medical Centre Nijimegen [homepage on the Internet].DAS.[cited 2007 October]. Available from http://www.das-score.nl/

3 Van Gestel AM.et al. Validation of rheumatoid arthritis improvement criteria that include simplified joint counts. Arthritis Rheum 1998, 10:1845-50.
