# Supplementary material for: Effectiveness of drug safety measures for reducing the incidence of adverse drug reactions: Post-hoc analysis of data from all-case surveillance of iguratimod using generalized estimating equations
Source: PLoS One. 2021 Jul 30;16(7):e0253513. doi: 10.1371/journal.pone.0253513 (PMC8323896; doi:10.1371/journal.pone.0253513)
Supplement: S2 File — (PDF) [file pone.0253513.s003.pdf]

Study title: Investigation of the Safety and Efficacy of Iguratimod in Patients with Rheumatoid Arthritis (All-case Surveillance Study)

1. PURPOSE OF THE STUDY

To investigate the safety and effectiveness of the administration of Iguratimod (CRM01T) for 52 weeks in patients with rheumatoid arthritis in clinical practice.

SAFETY SPECIFICATION

- Blood disorder
- Liver disorder
- Renal disorder
- Gastrointestinal disorder
- Idiopathic interstitial pneumonia
- Infection

2. Sample Size and Rationale

2.1 Sample size

2000 patients

2.2 Rationale

Register all patients where this drug has been used since its launch. An interim analysis is conducted using case report form 1 data (from the start of administration to 24 weeks), which is the planned sample size, and a request form for release of approval conditions is submitted to the Ministry of Health, Labor and Welfare (Japanese authority). Until the approval conditions are canceled, patient registration will continue, and case report form will be collected as necessary. In addition, for patients who have been administered for more than 24 weeks, a case report form 2 (from 25 weeks to 52 weeks later) will be collected and conducted analysis.

3. Patients for This Study

3.1 Target patients

Patients with rheumatoid arthritis and who take iguratimod.

3.2 Exclusion criteria

The following patients will be excluded from this study:

(1)Participants registered for this study previously

(2)Pregnant women or women suspected of being pregnant

(3)Participants with a serious liver disorder

(4)Participants with peptic ulcer

(5)Participants with known hypersensitivity to iguratimod or any of its excipients

3.3 Dosage and Administration

The usual adult dosage for oral use of 25 mg tablet of iguratimod once daily for 4 weeks or more, after which the dosage should be increased to one 25 mg tablet taken twice daily.

4. Number of Sites Participating in the Study and Specialties

All sites that use iguratimod will be investigated. Use of iguratimod is limited to sites and physicians who fulfill the following requirements and have concluded the contract for this study.

(1)Site requirements

- Can cooperate with this all-cases surveillance study and contract.
- Capable of specialized treatment for rheumatoid arthritis (Clinical trial participating sites, educational institutions certified by the Japanese Society of Rheumatology, training sites for specialists in the Japanese Orthopedic Association, etc.)
- Where sufficient treatment can be taken in an emergency.

(2)Physician requirements

- Investigator of clinical trial for iguratimod.
- Board certified rheumatologist of the Japan College of Rheumatology
- Board certified rheumatologist of the Japanese Orthopaedic Association
- Registered physician of the Japan Rheumatism Foundation

5. Study Methods

All-case surveillance with central registration will be employed for the study.

5.1 Study Request/Contract

The target sites are medical institutions that prescribe this drug (including external prescriptions). Our medical representatives will carefully explain the purpose of the study, the target patients, and the study methods to the investigators at the target sites. If an investigator approves participation in the study, the medical representative will proceed to conclude the contract. The contract must be concluded in writing between the head of the medical institution and the sponsor.

5.2 Selection of Target Patients and Registration

(1)Register all patients who use this drug from the date of launch of this drug until the approval conditions are released.

(2)The physician in charge of the study will register the necessary items on the registration form after the start of administration of this drug, and will FAX the from to the registration center.

5.3 Observation period

The observation period is 52 weeks for each patient.

5.4 Completion and submission of the case report form

The two forms shown below will be used for the study:

Case report form (1) – From the start of administration to 24 weeks

Case report form (2) – From 25 weeks to 52 weeks later

After the observation period is completed, the investigator must describe every study item for all the registered cases in the case report form and submit it to Medical Representable.,

6. Study Implementation Schedule

Survey period: Release approval condition or end of observation period (52 weeks), whichever is later

Registration will continue until the approval conditions are lifted by the Ministry of Health, Labor and Welfare.

7. Survey Items, etc.

7.1 Registration Form

The patient's initials, sex, date of birth (or age), patient identification number, date of initial administration, diagnosis, history of rheumatoid arthritis (duration, Steinbrocker functional classification[1], Steinbrocker disease classification[1], whether the patient fulfills any contraindications, and whether the patient fulfills any criteria for careful administration.

7.2 Case Report Form

(1) Patient characteristics

Body weight, height, inpatient/outpatient status, surgery history for rheumatoid arthritis, anti-CCP antibody, rheumatoid factor, complications, medical history, and drug allergy history.

(2) Administration of this drug

Daily dose (dose per administration and daily dosing frequency), dosing period (administration start date and administration stop date), and reasons for changes of dosage and administration.

(3) Concomitant drugs for rheumatoid arthritis

Presence/absence of concomitant drugs, names of concomitant drugs, administration period for concomitant drugs, route of administration, and daily dose.

(4) Concomitant drugs for other disease

Presence/absence of concomitant drugs, names of concomitant drugs, administration period for concomitant drugs, route of administration, and daily dose.

(5) Combination therapy for rheumatoid arthritis

Presence/absence of combination therapy, name of the therapy.

(6) Treatment course

Number of tender and swollen joints, ESR, CRP, patient's VAS, physician's VAS.

(7) Laboratory tests prior to initial administration of this drug relating to Safety specifications

Whether laboratory tests were carried out, the test date, and test results.

Laboratory tests specifically related to AEs are performed after initial administration of this drug.

Test items

Hematology tests:

Red blood cell count, hemoglobin level, hematocrit level, white blood cell count, neutrophil count, lymphocyte count, platelet count, etc.

Blood biochemistry tests:

AST(GOT), ALT(GPT), γ-GTP, Al-P, LDH, gamma-GTP, total bilirubin, serum albumin

Urinalysis:

Protein, glucose, and occult blood

Idiopathic interstitial pneumonia tests:

KL-6

(8) Effectiveness[EULAR DAS28][2,3]

The clinical effect is determined according to the following procedure.

1)DAS28 calculation

Calculated DAS28 using from a) Evaluation of joints (number of tender joints, number of swollen joints), b) ESR or CRP, c) Calculated from the patient's general evaluation (VAS) using a formula. DAS28 at the time of measurement is classified as follows.

1. DAS ≤ 3.2, 2. 3.2 <DAS ≤ 5.1, 3. DAS> 5.1

2)DAS28 improvement

The difference of DAS28 at each judgment is calculated from DAS28 at the start of administration, and the degree of DAS28 improvement is classified as follows.

1. Difference > 1.2, 2. 0.6 <Difference ≤1.2, 3. Difference ≤ 0.6

3)Clinical effect evaluation by DAS28

The clinical effect is determined in the following three stages based on (i) DAS28 calculation evaluation at each judgment and (ii) the effect determination based on the EULAR DAS28 improvement degree from the DAS28 improvement degree.

1. Effective, 2. Slightly effective, 3. Ineffective

(9) Adverse Events (AEs)

An AE refers to any untoward or unintended illness, condition, symptom, or abnormal change of laboratory test results in a patient administered this drug and does not necessarily have a causal relationship with the drug. Among AEs, events for which a causal relationship with this drug cannot be ruled out are defined as ADRs.

The following items will be investigated for AEs: (In principle, any AE should be followed up until the investigator considers it is no longer clinically important.)

Presence/absence of AEs, AE term, date of onset, severity, seriousness, handling of this drug after the event developed, treatment of the AE, laboratory tests related to the AE, date of outcome assessment (or outcome confirmation date), outcome, causal relationship with this drug, and possible factors other than this drug.

(10) Interaction with anticoagulants including warfarin

1)Warfarin combination subject

Presence or absence of bleeding events, detailed information on the occurrence of bleeding events, warfarin dosage, and blood coagulation clinical laboratory values before and after combination with this drug (PT-INR).

2)Patients with anticoagulants other than warfarin

Presence or absence of bleeding events, detailed information on the occurrence of bleeding events, dosage of anticoagulant, blood coagulation clinical laboratory values before and after combination with this drug (PT-INR, APTT, etc.).

7.3 Study schedule

CRF volume no.	Registr at-ion form	CRF (1)			CRF (2)	
Time / Item	Baseline	Week 2 to Week 8 of administration	Week 12 to Week 20 of administration	Week 24 of administration or at the time of cessation	Week 25 to Week 48 of administration	Week 52 of administration or at the time of cessation
Patient background	X					
Administration of this drug	◄—	—	—	—	—	—►
Concomitant drug for RA	◄—	—	—	—	—	—►
Concomitant drug for other disease		◄—	—	—	—	—►
Combination therapy for RA		◄—	—	—	—	—►
Treatment course	X	X	X	X	X	X
Safety specification	X	X	X	X	X	X
AEs		◄—	—	—	—	—►

CRF: Case report form, RA: Rheumatoid arthritis, AE: Adverse event

8. Methods of Analysis

8.1 Handling of case

The following criteria will be used for case rejection, judgment, and adverse events. If there is any matter that has not been agreed, the case study meeting will decide how to handle it.

8.1.1 Safety judgment

The following cases are excluded from the safety analysis.

(1)Patients who have not been administered this drug.

(2)Patients who did not visit after the first prescription date.

(3)Patients that have been registered in this study in the past.

8.1.2 Efficacy judgment

The following cases are excluded from the effectiveness analysis for safety analysis.

(1)Non-target disease cases.

(2)Patients missing required items for DAS28 calculation at the start of administration.

(3)Patients who have been treated with this drug before the start of the study (continued from post-marketing clinical trials)

8.1.3 Other handling standards

The detailed data period is specified in the statistical analysis plan.

8.2 Analytical items

8.2.1 Patient composition

The number of sites, the number of registration, the number of collected CRFs, the number of safety analysis population, the number of effectiveness analysis population, etc.

8.2.2 Safety items

The incidence of ADRs, the incidence of each patient subgroup, the incidence of serious ADRs, the incidence of Aes, the incidence of serious Aes, the list of AE emerged patients, etc.

8.2.3 Effectiveness items

DAS28, treatment effect for each patient subgroup, etc.

8.2.4 Safety specification

Blood disorder, liver disorder, renal disorder, gastrointestinal disorder, idiopathic interstitial pneumonia, infection.

8.2.5 Other items concerning patients with special background

Patient with liver disorder, patient with renal disorder, children, elderly, underweight (< 40 kg)

8.3 Analytical method

When performing comparisons, perform appropriate analysis according to the item from methods such as $\chi 2$ test, Fisher's exact test, t-test, etc.

On the other hand, when examining the background factors that affect the safety and effectiveness of this study, logistic regression analysis and the like will be performed.

8.3.1 Safety analysis

(1)For the side effects, calculate the number and rate of occurrence of cases, and the number and rate of occurrence by type. For serious side effects, the number of cases and the rate of occurrence, and the number and number of occurrences by type are calculated.

(2)To investigate factors that may affect safety, calculate the incidence of adverse reactions by patient background, and perform the $\chi 2$ test or Fisher's exact test. The significance level is 5% on both sides.

(3)For adverse events, calculate the number and rate of occurrence of cases, and the number and incidence of cases by type. In addition, for serious adverse events, the number of cases and the rate of occurrence, and the number and number of occurrences by type are calculated.

8.3.2 Effectiveness analysis

(1)For clinical effectiveness evaluation, cases judged as "effective" or "moderately effective" in the DAS28 improvement degree of EULAR are counted as valid.

(2)To examine the factors that may affect the efficacy, calculate the efficacy rate for each patient background, and perform the $\chi 2$ test or Fisher's exact test. The significance level is 5% on both sides.

8.3.3 Other analysis

To classify renal dysfunction, the creatinine clearance CLcr (mL / min) is calculated from the serum

creatinine and body weight at the start of administration using the Cockcroft-Gault formula, and classified to normal renal function (CLcr ≥ 80), mild renal dysfunction (50 ≤ CLcr <80), moderate renal dysfunction (30 ≤ CLcr <50)) or Severe renal dysfunction (CLcr <30) based on FDA guidance (Guidance for Industry: Pharmacokinetics in Patients with Impaired Renal Function –Study Design, Data Analysis, and Impact on Dosing and Labeling).

9. Study Organizational Structure

The organizational structure is provided in the Risk Management Plan.

10. OTHER REQUIREMENTS

(1) Protocol revisions

If new findings are obtained during the implementation period of this study, whether revisions should be made to the protocol will be discussed, and it will be revised as necessary. If a partial change, etc. of dosage and administration/indications is approved, whether revisions should be made to the protocol will be discussed, and it will be revised as necessary.

(2) Actions to be taken for issues or inquiries

Implementation of a special drug use-result study and/or post-marketing clinical study will be discussed in the following cases in order to detect or confirm relevant factors and verify estimates, etc. obtained as a result of this investigation:

1) If a serious safety concern has been raised, such as occurrence of unexpected/serious ADRs.
2) If there is an evident increase in the incidence of a serious ADR.
3) If concern about safety and effectiveness is raised by comparison with data obtained prior to approval.

Reference

1 Steinbrocker O.et al.Therapeutic criteria in rheumatoid arthritis.JAMA140:659, 1949

2 Department of rheumatology, University Medical Centre Nijimegen [homepage on the Internet].DAS.[cited 2007 October]. Available from http://www.das-score.nl/

3 Van Gestel AM.et al. Validation of rheumatoid arthritis improvement criteria that include simplified joint counts. Arthritis Rheum 1998, 10:1845-50.
